# Research and Remedies

**Published:** 1914-04-11

**Authors:** 


### RESEARCH AND REMEDIES.

### The Cause of Whooping-Cough.

At the end of last century Bordet and Gengou described a minute bacillus as the probable organism responsible for that troublesome infantile disease, whooping-cough. Their work was in course of time generally accepted as sound; but the difficulties presented by investigation of the matter rendered further research into the question not very profitable. Lately Drs. Mallory, Hornor, and Henderson have taken up the matter anew; having been fortunate enough to encounter two cases in which death had taken place quite early in the disease, they were enabled to study the changes in the bronchial mucous membrane before secondary infections had been implanted on it. They find that whooping-cough is due to a minute bacillus which occurs in large numbers between the cilia of the epithelial cells lining the trachea and bronchi, and possibly also the nose. The location of the organism is characteristic of this disease; and its action is probably mainly mechanical, for it interferes with the normal movements of the cilia, and possibly leads to their destruction. This effect prevents the proper removal of secretion, and results in a continuous irritation, which causes coughing and the characteristic spasms of whooping. The bacillus is that first described by Bordet and Gengou, and is the primary cause of the disease, not a secondary invader as some authorities have thought. With pure cultures of this organism, the three Boston workers have produced in young animals (puppies, rabbits) the same characteristic lesion of the mucous membrane as is found in man. In four instances (one puppy, three young rabbits) they were able to obtain the organism again in pure culture. Further, sub-cultures from the original bacillus isolated by Bordet himself have also produced the same lesions in animals, and this bacillus, too, has been recovered in pure culture. These results, they think, should encourage further investigation and search for a vaccine or anti-toxin to combat the disease. The usual natural tendency to recovery and the disappearance of the bacilli in the course of a few weeks suggest that the conditions for growth become unfavourable, probably through the development by the patient of some anti-body. The details of these researches, some of which had been previously published, are to be found in "The Medical and Surgical Reports of the Boston City Hospital," of which a new series has just appeared after an interval of eight years.

### The Ideal Dentifrice.

According to Mr. P. Furnivall, the best dentifrice for preserving the teeth and keeping both them and the gums quite healthy is a mixture of lysol, three parts, and chloroform one part. To begin with, fifteen drops of this mixture should be used in half a pint of (sterile) water; after a short time, when the gums have got used to this strength, it can be used with advantage in more concentrated solution, up to a drachm in half a pint. To get the full value, each mouthful should be held in the mouth for about twenty seconds, then well brushed over the gums and tongue, and the mouth finally rinsed with sterile water. Other points in oral hygiene must be attended to properly, such as the cleansing and drying of the toothbrush after use, and the use of the silk thread to get food débris out of the teeth. Ordinary tap-water is so comparatively free from micro-organisms that the emphasis laid upon the use of sterile water seems unnecessary and meticulous; however, Mr. Furnivall insists on the point, though we fear it is a counsel of perfection.

